# ALPINE: a scalable pipeline for comprehensive classification of gene-editing outcomes from long-read amplicon sequencing

**DOI:** 10.1093/bioinformatics/btag528

**Published:** 2026-07-20

**Authors:** Yu Chen, Xing-Huang Gao, Athea Vichas, Jianbin Wang, Ryan Golhar, Isaac Neuhaus

**Affiliations:** Research and Development, Bristol-Myers Squibb Company, Princeton, NJ 08540, United States; Product Development & Supply, Bristol-Myers Squibb Company, Seattle, WA 98109, United States; Product Development & Supply, Bristol-Myers Squibb Company, Seattle, WA 98109, United States; Product Development & Supply, Bristol-Myers Squibb Company, Seattle, WA 98109, United States; Research and Development, Bristol-Myers Squibb Company, Princeton, NJ 08540, United States; Research and Development, Bristol-Myers Squibb Company, Princeton, NJ 08540, United States

## Abstract

**Summary:**

CRISPR genome editing has enabled precise genetic modification for gene and cell therapies, but edits often produce heterogeneous on-target outcomes, including homology-directed repair (HDR) knock-ins, DNA repair template integrations, and structural variants. Existing tools are frequently limited to short reads or lack viral vector-specific integration categories needed for therapeutic development. Here, we present ALPINE (Amplicon Long-read Pipeline for INtegration Evaluation), a scalable and reproducible pipeline for classifying and quantifying gene-editing outcomes from long-read amplicon sequencing supporting both PacBio HiFi and Oxford Nanopore platforms. ALPINE classifies reads into 10+ categories, including DNA repair vector integration subtypes, and performs variant calling near the gene-edited site with batch, multi-sample reporting. Uniquely, ALPINE can distinguish between cells treated with multiple DNA repair vectors and identify distinct molecular features, such as inverted terminal repeats (ITRs), enabling comprehensive characterization of complex gene editing outcomes. Dual-target benchmarking on simulated datasets demonstrated high accuracy for transgene integration events. Independent validation on public crosslinked-HDR dataset confirmed ALPINE’s integration detection capabilities, and application to edited T cell samples demonstrated comprehensive gene-editing outcome profiling.

**Availability:**

ALPINE is available under MIT license at https://github.com/Maggi-Chen/ALPINE and https://doi.org/10.5281/zenodo.20272510. All analysis scripts and visualization code used in this manuscript are available at https://github.com/Maggi-Chen/ALPINE-manuscript-analysis. Simulated datasets are deposited at Zenodo (https://doi.org/10.5281/zenodo.20260865). Public dataset PRJNA913199 is available through NCBI SRA.

## 1 Introduction

CRISPR-Cas genome editing has revolutionized cell-based therapies by enabling precise genetic modifications, such as gene knockouts and transgene knock-ins through homology-directed repair (HDR) with viral vector donors (Doudna and Charpentier 2014). However, repair of CRISPR-induced double-strand breaks can yield heterogeneous outcomes, including structural variants and viral vector integrations. While recent studies have illustrated that in addition to intended perfect HDR, gene editing can result in additional repair outcomes, including large deletions and DNA repair template integration (e.g. AAV vector sequences integrated at the cleavage site) (Hunt *et al.* 2023). In adeno-associated virus (AAV)-mediated editing, approximately 1–2% of reads contain AAV integrations, including full-length AAV genomes and fragments with inverted terminal repeat (ITR) sequences (Simpson *et al.* 2023). Such unintended modifications at gene-edited loci (Fig. S1, available as supplementary data at *Bioinformatics* online) can disrupt transgene expression, compromise efficacy, or introduce safety risks, making accurate characterization critical for regulatory evaluation of gene-edited therapies.

Despite the importance of capturing gene-editing outcomes, existing tools have major limitations for long-read amplicon data from Cas-mediated editing. CRISPResso2, one of the most widely used genome editing analysis tools, is designed for short-read Illumina data with maximum practical read length at ∼600 bp, limiting detection of large structural variants, full-length HDR knock-ins, and multi-kilobase AAV integrations (Clement *et al.* 2019). The knock-knock pipeline supports long reads and profiles HDR outcomes (Canaj *et al.* 2019), but lacks AAV-specific categories (e.g. integration with/without ITR) and supports only a single homologous donor template per target site, preventing attribution when multiple AAV vectors may integrate at the same locus. Its multi-aligner workflow (BLASTn, STAR, minimap2) also increases computational overhead (Canaj *et al.* 2019). As a result, researchers often rely on manual counting of AAV integrations from long-read alignments (Simpson *et al.* 2023).

To address these limitations, we developed ALPINE (Amplicon Long-read Pipeline for INtegration Evaluation), a scalable bioinformatics pipeline for automated classification and quantification of gene-editing outcomes from long-read amplicon sequencing data. ALPINE supports both Pacific Biosciences High-Fidelity (PacBio HiFi) and Oxford Nanopore Technologies (ONT) sequencing platforms with platform-specific optimizations. ALPINE classifies reads into 10+ variant categories, including HDR knock-ins and AAV vector insertions with or without ITR sequences, and supports multiple AAV vectors to identify which vector contributed each integration event (Supplementary Note 1, available as supplementary data at *Bioinformatics* online). ALPINE also performs variant calling within a defined window around the cleavage site. Built with Docker containers and CWL workflows, ALPINE enables reproducible, cloud-deployed analysis suited to high-throughput studies and regulatory environments.

## 2 Methods

### 2.1 Workflow overview

**Figure 1:**
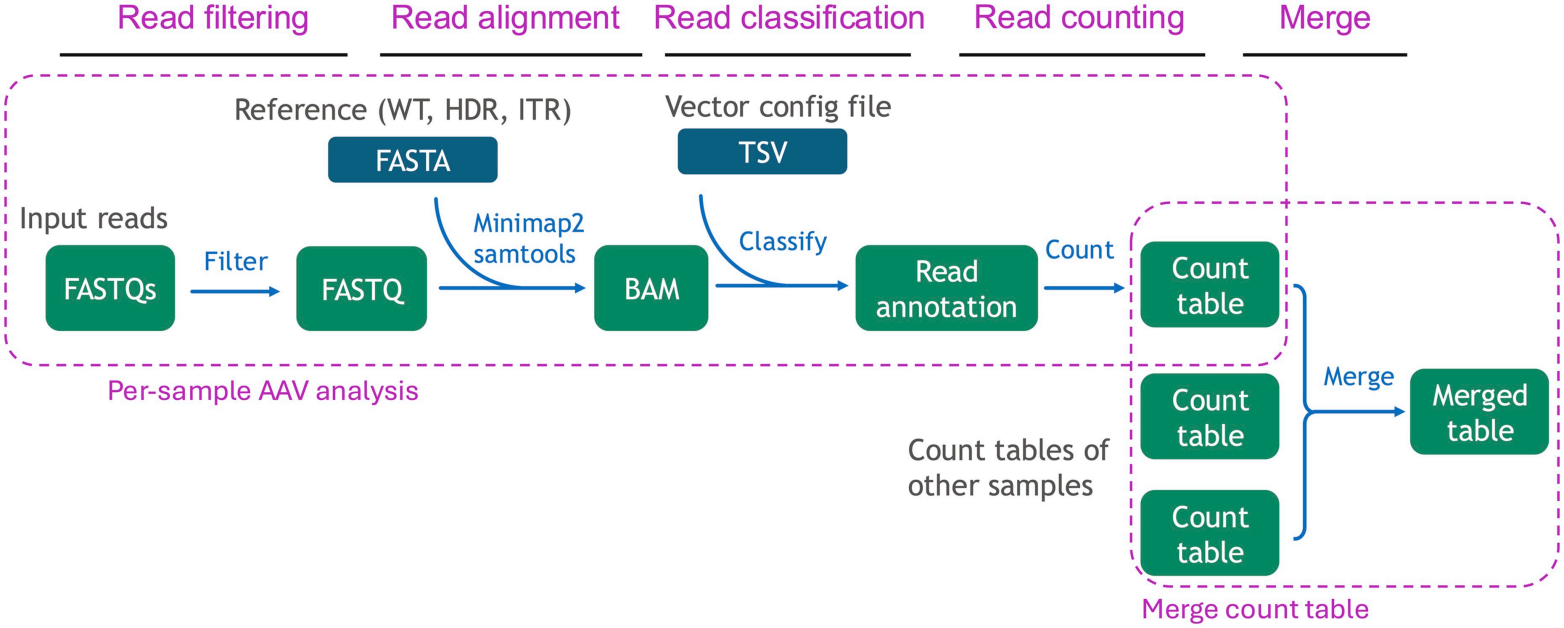
Overview of the ALPINE pipeline for long-read amplicon sequencing analysis. ALPINE workflow includes five major steps: read filtering, alignment with minimap2, read classification, read counting, and table merging.

The ALPINE pipeline uses a multi-cloud launcher framework to execute a per-sample analysis workflow and merge per-sample outputs into a final summary table. Each workflow consists of five sequential steps: read filtering, alignment, classification, counting, and merging (Fig. 1). The inputs include FASTQ files, reference sequences (wild-type, HDR, and optionally AAV integrations), a configuration file specifying cleavage site coordinates and vector element boundaries (ITRs and transgene), primer and homology arm sequences, and optional quality thresholds. Outputs include per-sample classification tables, quality control reports, and a merged summary table. Key analysis parameters of ALPINE are configurable, with default values derived from established conventions and empirical optimization in diverse CRISPR editing datasets.

### 2.2 Read filtering and alignment

Reads are first filtered to ensure completeness and sequencing quality. Primer sequences are searched at both read ends (default 50 bp window) to confirm complete amplicon sequencing. Reads containing both primers are retained. Read average sequencing quality is calculated and reads below the threshold (default Q30 for PacBio HiFi, Q10 for ONT) are filtered from downstream analysis. A quality control report with histogram plots of read length and quality distributions before and after filtering is generated (Fig. S2, available as supplementary data at *Bioinformatics* online).

Filtered reads are aligned to reference sequences using minimap2 with the map-hifi preset for PacBio HiFi data or map-ont preset for ONT data (Li 2018). The reference FASTA includes WT sequence, HDR knock-in sequence, and AAV vector integration sequences. Additional references can be included to capture other expected editing outcomes (e.g. antisense transgene knock-in). The config file provides 1-based coordinates for WT cleavage site, HDR transgene boundaries, and ITR boundaries in the AAV integration reference. Aligned reads are sorted and indexed using SAMtools (Li *et al.* 2009).

### 2.3 Read classification

Reads are classified based on alignment results (Fig. S3, available as supplementary data at *Bioinformatics* online). Classification involves a series of alignment and re-alignment steps:

- Reads aligned to WT reference: Variant calling is performed within a defined window (default ±20 bp) around the cleavage site, assigning each read to one of the variant types: unmodified, DEL-large (≥50 bp), DEL-small (<50 bp), INS-large (≥50 bp), INS-small (<50 bp), unmodified-with-SNP, INV (inversion), DUP (duplication). Large insertions are extracted and re-aligned to all references to determine whether inserted sequences originate from transgene or DNA repair template (e.g. AAV vector).
- Reads aligned to HDR/AAV integration references: Reads aligned to HDR or AAV references are evaluated for transgene and AAV content to distinguish between perfect HDR knock-in and AAV integration (referred as NonHDR). AAV insertions are further classified into NonHDR-with-ITR and NonHDR-without-ITR categories based on ITR sequence presence.
- Re-alignment modules: Three re-alignment modules address imperfect initial alignments using platform-specific minimap2 presets. (1) Inserted-sequence re-alignment for WT-aligned reads with large insertions (≥50 bp), extracting inserted sequences and re-aligning to HDR/AAV references. (2) Clipped-sequence re-alignment for WT-aligned reads with unmapped clipped segments (≥100 bp), extracting clipped sequences and re-aligning to check for transgene content and call variants. (3) WT re-alignment for reads aligned to HDR/AAV references that lack transgene sequence, re-aligning full read to WT reference for variant calling.
- False-negative rescue: A second re-alignment module (“patcher”) re-analyzes unclassified reads by realigning them to the WT reference using minimap2 v2.16 with map-pb preset, allowing detection of large deletions missed in initial classification. Reads matching large deletion pattern are rescued and reassigned to DEL-large.

Additional details and pseudocode for classification are provided in Supplementary Note 2 and 3, available as supplementary data at *Bioinformatics* online.

### 2.4 Read counting and merging

Following read classification, ALPINE quantifies the number of reads within each variant category for each sample, and outputs the results as a count table. A pie chart is generated to visualize category proportions (Fig. S4, available as supplementary data at *Bioinformatics* online). When multiple samples are processed, count tables are merged into a combined summary table (Fig. S5, available as supplementary data at *Bioinformatics* online). For experiments involving different target sites or transgenes, the merged table includes all vector-specific categories with zero filling for categories not applicable to a sample.

### 2.5 Cloud integration and scalability

ALPINE is implemented using CWL and Docker containers for platform agnostic deployment. Each step runs in an isolated Docker container. A multi-platform launcher manages platform-specific configuration and resource allocation, supporting SevenBridges Genomics, Amazon HealthOmics and Arvados. This architecture enables scalable analysis of large datasets while maintaining reproducibility required for regulatory submissions.

## 3 Results

### 3.1 Benchmark on simulated datasets

To evaluate classification accuracy, we performed simulation benchmarking for ALPINE and knock-knock on dual-target gene editing datasets (Supplementary Note 4, available as supplementary data at *Bioinformatics* online). Simulated datasets included transgene integration events (HDR, ITR-mediated, truncated HDR, and cross-target contamination) and endogenous variants (deletions, insertions, SNPs, and wild-type sequences) for both TRAC and TRBC target sites, with PacBio HiFi and ONT reads generated by Badread (Wick 2019). For transgene integration events, ALPINE achieved overall F1-score of 98.95% on PacBio HiFi and 99.63% on ONT data, while knock-knock reached 97.59% on PacBio HiFi and 88.67% on ONT data (Fig. S6, Tables S1–S4, available as supplementary data at *Bioinformatics* online). ALPINE was particularly robust in integration event classification, maintaining high precision when distinguishing between different integration types. For endogenous variants, ALPINE achieved F1-scores above 95% for large indels in both platforms. However, both tools showed reduced performance for small indels, SNPs, and wild-type sequences, likely reflecting the difficulty of distinguishing true low-level variants from sequencing errors inherent to long-read technologies.

### 3.2 Evaluation on real-world crosslinked template dataset

To evaluate ALPINE’s performance on real-world gene editing data, we analyzed a publicly available dataset that investigates effects of crosslinked homologous repair templates on CRISPR-Cas9 editing efficiency (Ghasemi *et al.* 2023). We applied ALPINE and knock-knock to PacBio data, and CRISPResso2 to Illumina data from K562 cells edited at the HBB locus across a range of experimental conditions, including unmodified, untreated, UV-only controls, and cells treated with varying psoralen dosages (Supplementary Note 5, available as supplementary data at *Bioinformatics* online).

ALPINE analysis of the PacBio data revealed editing outcome distributions consistent with experimental conditions, where unmodified samples showed an average editing rate of 8.32%, while modified samples demonstrated consistently higher editing rate ranging from 75.49% to 89.24% (Fig. S7, available as supplementary data at *Bioinformatics* online). Across untreated, UV-only, and psoralen-treated conditions, ALPINE and knock-knock reported comparable editing rates, in agreement with CRISPResso2 results showing no significant differences between psoralen treatment groups and UV-only controls—consistent with prior reports that crosslink treatment does not alter non-homologous end-joining outcomes. Tool concordance analysis between ALPINE and knock-knock was highly consistent across all 34 samples, with strong correlations in read proportions for indel and wild-type outcome classes (Fig. S8, available as supplementary data at *Bioinformatics* online). One notable difference was that, ALPINE classified fewer reads as unclassified compared to knock-knock’s uncategorized reads, suggesting more comprehensive outcome classification of ALPINE. We further analyzed the detection of a characteristic 9 bp deletion expected in ∼25–33% of edited HBB sequences (Ghasemi *et al.* 2023). Both ALPINE and knock-knock consistently detected this deletion at expected frequencies across all modified conditions (Fig. S9, available as supplementary data at *Bioinformatics* online).

For plasmid integration events, ALPINE outperformed knock-knock, achieving higher sensitivity in psoralen-treated groups and lower false-discovery rate in unmodified samples in which no plasmid integration is expected (Fig. S10, available as supplementary data at *Bioinformatics* online). Manual inspection of reads classified as integration events, by both tools and by ALPINE only, confirmed presence of donor sequence in reads of varying length via alignment analysis (Fig. S11, available as supplementary data at *Bioinformatics* online). Collectively, these results establish ALPINE as an accurate and comprehensive tool for classifying the full range of outcomes in complex gene editing experiments, with superior advantages in integration event detection.

### 3.3 Application to gene-edited T cell samples

We applied ALPINE to PacBio HiFi amplicon sequencing data from five edited human T cell samples, each edited at two distinct genomic loci using CRISPR ribonucleoprotein and AAV-delivered transgene constructs, generating 10 datasets in total. ALPINE successfully classified reads across all samples, revealing consistent patterns where HDR knock-in events predominated while structural variants and non-HDR integrations occurred at lower frequencies at both target sites (Table S5, available as supplementary data at *Bioinformatics* online). Read-length distributions correlated strongly with ALPINE’s quantitative classifications, with samples showing higher knock-in frequencies displaying higher peaks in the knock-in–associated read-length range, and those with higher knockout frequencies showing correspondingly enhanced knock-out peaks (Fig. S12, available as supplementary data at *Bioinformatics* online). This concordance between read-length patterns and ALPINE’s algorithmic classifications validates ALPINE’s accuracy for comprehensive gene-editing outcome quantification in clinical research settings.

### 3.4 Computational performance analysis

ALPINE demonstrated superior computational efficiency compared to knock-knock, achieving 3-fold faster processing on simulated data and 1.7-fold speedup on real-world datasets (Table S6, available as supplementary data at *Bioinformatics* online). Notably, ALPINE’s architecture is optimized for cloud-based high-throughput analysis. Using a multi-platform launcher, ALPINE processed all 34 samples in parallel on the SevenBridges cloud platform in under 11 minutes, demonstrating exceptional scalability for high-throughput genomic studies that require rapid analysis of large sample cohorts.

## 4 Discussion and conclusions

Compared with existing tools, ALPINE provides automated, end-to-end quantification of on-target gene-editing outcomes from long-read am-plicon sequencing and is configurable for both viral and non-viral do-nor methods. ALPINE classifies reads into “donor-aware” categories (including AAV integration subtypes when ITR/transgene boundaries are provided) and supports multi-vector experiments by attributing each integration event to the corresponding vector. To balance classification accuracy with scalability, ALPINE combines fast minimap2 alignment with targeted re-alignment of ambiguous reads, reducing unnecessary computational overhead without sacrificing precision. The result is a tool that produces standardized, auditable outputs well-suited for both high-throughput research and regulatory workflows.

Accurate classification of complex editing outcomes requires more than standard read alignment, particularly when reads contain large insertions, soft-clipped segments, or structural variants that can cause misclassification. To address this, ALPINE incorporates a set of target-ed re-alignment modules that act on reads flagged as potentially mis-classified. For example, when a wild-type-aligned read contains a large insertion, ALPINE extracts that inserted sequence and re-aligns it against HDR and AAV references for detection of transgene content. Similarly, reads with large soft-clipped segments undergo clipped-sequence re-alignment to recover donor or vector-derived sequence that standard alignment would otherwise discard. Lastly, a dedicated “patcher” module performs a secondary minimap2 alignment pass on initially unclassified reads, rescuing large deletions that may be missed in the initial classification step. Together, these modules systematically reduce misclassification, improving both sensitivity and classification completeness.

In conclusion, ALPINE provides an automated, cloud-deployable pipe-line for classification and quantification of gene-editing outcomes from long-read amplicon sequencing data, including detailed characteriza-tion of AAV integration events. ALPINE addresses a key gap in auto-mated analysis of long-read AAV integration outcomes and enables vector-specific and regulatory relevant assessment of complex editing outcomes for gene and cell therapy research.

## Supplementary Material

btag528_Supplementary_Data

## Data Availability

ALPINE is available under MIT license at https://github.com/Maggi-Chen/ALPINE and https://doi.org/10.5281/zenodo.20272510. All analysis scripts and visualization code used in this manuscript are available at https://github.com/Maggi-Chen/ALPINE-manuscript-analysis. Simulated datasets are deposited at Zenodo (https://doi.org/10.5281/zenodo.20260865). Public dataset PRJNA913199 is available through NCBI SRA.
