# Supplementary material for: ALPINE: a scalable pipeline for comprehensive classification of gene-editing outcomes from long-read amplicon sequencing
Source: Bioinformatics. 2026 Jul 20;42(7):btag528. doi: 10.1093/bioinformatics/btag528 (PMC13395072; doi:10.1093/bioinformatics/btag528)
Supplement: btag528_Supplementary_Data [file btag528_supplementary_data.docx]

**Supplementary Information**

**Supplementary Note 1: ALPINE Output Classification Categories**

ALPINE classifies each sequencing read into one of the following categories based on the detected genomic alterations (Numerical thresholds mentioned below represent default settings and are mostly configurable parameters):

- **Unmodified Outcomes**

**Unmodified**: Reads that align perfectly to the wildtype reference sequence with no detectable variants within the variant calling window. These represent cells that were not edited by the CRISPR system.

**Unmodified-with-SNP**: Reads that contain only single nucleotide polymorphisms (SNPs) or substitutions compared to the reference. These likely represent natural genetic variation or sequencing errors rather than CRISPR-induced modifications.

- **Transgene Integration Events**

**HDR**: Homology-directed repair events where the AAV vector template has been successfully integrated with high fidelity. These reads show coverage of HDR template sequence with high sequence identity to the expected template, without (or <10bp of) ITR sequence integration. These represent the desired precise gene editing outcome.

**Non-HDR-with-ITR**: Vector integration events that contain AAV inverted terminal repeat (ITR) sequences (>10bp coverage) but lack proper HDR template integration. These represent partial or imprecise vector integration events.

**Non-HDR-without-ITR**: Vector integration events that show evidence of AAV vector sequences without ITR content, but lack proper HDR template integration (identity <98% for PacBio HiFi, <90% for ONT) or have truncated HDR integration (transgene length <99% for PacBio HiFi, <95% for ONT). These represent imprecise integration events.

- **CRISPR-Induced Variants**

**DEL-small**: Small deletions (<50bp) at the target site, typically resulting from imprecise non-homologous end joining (NHEJ) repair of CRISPR-induced double strand breaks.

**DEL-large**: Large deletions (≥50bp) at the target site, representing more extensive NHEJ-mediated repair or loss of genomic material between multiple cut sites.

**INS-small**: Small insertions (<50bp) at the target site, usually resulting from NHEJ repair mechanisms that insert random nucleotides during double strand break repair.

**INS-large**: Large insertions (≥50bp) at the target site that do not match the provided AAV vector sequence. These may result from integration of contaminating DNA, complex rearrangements, or large NHEJ-mediated insertions.

**INV**: Inversions detected at the target site, where a genomic segment has been reversed in orientation, typically resulting from NHEJ repair between two CRISPR cut sites.

**DUP**: Duplications detected at the target site, where genomic segments have been duplicated, often arising from complex NHEJ repair mechanisms.

- **Unresolved Classifications**

**Unclassified**: Reads that could not be confidently classified into any of the above categories. These include unmapped reads, reads with complex alignment patterns, and reads not spanning cleavage site.

**Supplementary Note 2: ALPINE Read Classification Methods**

ALPINE employs a multi-step classification algorithm that assigns each sequencing read to a variant category based on its alignment to reference sequences. The algorithm includes three re-alignment modules to handle imperfect initial read alignments, ensuring comprehensive classification of diverse editing outcomes.

**Module 1: Insertion re-alignment.** For reads initially aligned to the wild-type (WT) reference that contain insertions ≥50 bp near the cleavage site (±20 bp by default), ALPINE extracts the inserted sequence and re-aligns it against all reference sequences (WT, HDR, and AAV integrant). If the inserted segment aligns to an HDR or AAV integrant reference, the read is re-evaluated for transgene content and reclassified accordingly. Conversely, if the inserted sequence aligns only to the WT reference or fails to align to any alternative reference, the read is assigned to the INS-large category.

**Module 2: Unmapped/clipped sequence re-alignment.** For reads aligned to the WT reference that contain unmapped or soft-clipped sequences ≥10

0 bp, ALPINE extracts these sequences and re-aligns them to the HDR and AAV integrant reference sequences. This module identifies cases where part of a read correctly maps to the WT genome while another portion contains transgene or vector-derived sequence that was not captured by the initial whole‑read alignment. Such mixed‑origin reads indicate potential integration events that would otherwise be missed, enabling more comprehensive detection and classification of complex repair outcomes.

**Module 3: HDR/AAV integrant aligned read re-evaluation.** For reads initially aligned to HDR or AAV integrant reference sequences that lack detectable transgene sequence—based on the expected transgene boundaries defined in the configuration file—ALPINE re-aligns the entire read to the WT reference for variant calling. This module addresses cases in which a read is preferentially aligned to the HDR or ITR reference due to sequence homology within the arms but does not actually contain transgene sequence. By re-evaluating such reads against the WT reference, ALPINE prevents misclassification and ensures accurate assignment of true editing outcomes.

**Patcher module (Large-Del false-negative rescue).** After the primary classification step is complete, ALPINE applies a post‑processing patcher module to recover potential false‑negative large‑deletion events. Reads initially categorized as Unclassified are re‑examined by extracting their sequences directly from the BAM file and re‑aligning them to the WT‑only reference using minimap2 v2.16. The patcher intentionally uses minimap2 v2.16 because for reads containing large deletions, newer releases (≥2.28) of minimap2 tend to align only sequences from one side of the deletion event and leave the other side unmapped, which can mask the deletion signatures. Large deletions (≥50 bp) are detected using both CIGAR-based analysis near the target site and segment-based analysis within a defined window. Only DEL‑large reads are rescued to maintain conservative reclassification, and the read name classification file is updated in place with the corrected assignments.

**Multi-vector classification.** When multiple AAV vectors are used within the same experiment—such as distinct AAV vectors delivering different transgenes to separate genomic loci—ALPINE assigns each integration event to the correct vector based on the specific reference sequence to which the read aligns. This enables vector-resolved quantification of editing outcomes even in complex multiplexed designs. The resulting count table contains per-vector columns for HDR, Non-HDR-with-ITR, and Non-HDR-without-ITR categories (e.g., HDR-VectorA, Non-HDR-with-ITR-VectorA, Non-HDR-without-ITR-VectorB). These vector-specific measurements allow researchers to independently track editing efficiency, integration profiles, and ITR retention for each vector at its corresponding target site.

**Supplementary Note 3:** **Pseudocode for classification**

CLASSIFICATION DECISION LOGIC:

FOR each aligned read:

1. PRIMARY ALIGNMENT CHECK:

IF read aligns to wildtype (WT) reference:

GOTO 2 (Wildtype Processing)

ELSE IF read aligns to HDR/ITR reference:

GOTO 3 (HDR/ITR Vector Classification)

ELSE IF read is unmapped:

GOTO 5 (Complex Alignment Classification)

2. WILDTYPE PROCESSING:

IF read contains soft-clipped sequences:

- Extract clipped sequence portions

- Re-align clipped sequences to HDR/ITR reference

IF clipped sequences align to transgene (HDR/ITR):

GOTO 3 (HDR/ITR Vector Classification)

ELSE:

GOTO 4 (Variant Classification)

ELSE:

GOTO 4 (Variant Classification)

3. HDR/ITR VECTOR CLASSIFICATION:

IF read aligns to HDR or ITR sequences:

IF ITR_coverage > 10bp:

IF HDR_coverage > 10bp:

IF HDR_sequence_match >= 98%:

Classification = "HDR"

ELSE:

Classification = "Non-HDR-without-ITR"

ELSE:

Classification = "ITR" (Non-HDR with ITR)

ELSE IF HDR_coverage > 10bp:

IF HDR_sequence_match >= 98%:

Classification = "HDR"

ELSE:

Classification = "Non-HDR-without-ITR"

ELSE:

Re-align to wildtype → GOTO 4 (Variant Classification)

4. VARIANT CLASSIFICATION:

IF read aligns to wildtype with variants:

IF no variants detected:

Classification = "Unmodified"

ELSE IF single nucleotide change:

Classification = "Unmodified-with-SNP"

ELSE IF insertion detected:

IF insertion_size >= 50bp:

- Extract insertion sequence

- Re-align insertion sequence to AAV reference

IF insertion_sequence aligns to AAV:

→ GOTO 3 (HDR/ITR Vector Classification)

ELSE:

Classification = "INS-large"

ELSE:

Classification = "INS-small"

ELSE IF deletion detected:

IF deletion_size >= 50bp:

Classification = "DEL-large"

ELSE:

Classification = "DEL-small"

ELSE IF inversion detected:

Classification = "INV"

ELSE IF duplication detected:

Classification = "DUP"

5. COMPLEX ALIGNMENT CLASSIFICATION:

IF read is unmapped or has complex alignment:

Apply patcher_rescue_algorithm()

IF large deletion recovered:

Classification = "DEL-large"

ELSE:

Classification = "Unclassified"

**Supplementary Note 4: Simulated Dataset Generation and Benchmark**

Simulated datasets were generated to benchmark performance of ALPINE and knock-knock across diverse genomic editing outcomes and sequencing platforms usingual-target simulation framework representing CAR-T cell engineering scenarios with simultaneous CD19→TRAC and CD22→TRBC knock-in targets. RefSeq cDNA sequences were obtained for CD19 (NM_001178098.2) and CD22 (NM_001771.4), with precise cleavage site coordinates extracted from Ensembl GTF files (TRAC: chr14:22,547,650, TRBC: chr7:142,791,900). Template sequences were constructed for 12 distinct categories per target encompassing the full spectrum of genome editing outcomes:

- HDR integrations: CD19 transgene integrated into TRAC locus and CD22 transgene integrated into TRBC locus via homologous recombination
- ITR-mediated integrations: Full AAV vector integrations with bilateral ITR sequences flanking transgenes at both target sites
- One-sided ITR integrations: AAV vector integrations retaining ITR sequences on either 5' or 3' side only
- Truncated HDR integrations: HDR templates with transgenes truncated at random positions (10-90% of full length) to simulate imperfect recombination events
- Vector cross-target - HDR: CD22 transgene integrated into TRAC locus and CD19 transgene integrated into TRBC locus, representing off-target vector delivery
- Vector cross-target - ITR integration: Same cross-target scenarios via ITR-mediated integration
- Wild-type sequences: Unedited target sequences with cleavage site intact
- SNP variants: Single nucleotide polymorphisms introduced near cleavage sites
- Small deletions: 1-50bp deletions affecting cleavage regions
- Large deletions: 51-200bp deletions spanning cleavage sites
- Small insertions: 1-50bp random sequence insertions near cleavage sites
- Large insertions: 51-200bp random sequence insertions near cleavage sites

For variant simulation (SNPs, deletions, and insertions), 500 templates per target per variant class were generated with diverse sizes and positions near cleavage sites. Template-based read simulation was performed using Badread with platform-specific error models: PacBio HiFi simulation employed “--identity 99.5,99.9,0.5 --error_model pacbio2021 --qscore_model pacbio2021” parameters, while Oxford Nanopore simulation used “--error_model nanopore2023 --qscore_model nanopore2023” parameters. Quality validation including PCR primer compatibility verification and minimum read length filtering (≥100bp) to eliminate simulation artifacts. Final datasets comprised 60,000 reads per platform, pooled and shuffled to create mixed populations.

ALPINE was run on simulated PacBio HiFi datasets with option “--min_qual 0 --data_type pacbio-hifi”, and was run on simulated ONT datasets with option “--min_qual 0 --data_type nanopore” to allow all reads to pass filtering step. Knock-knock v0.8.2 was run on the same simulated FASTQ datasets using the published process-sample command of its CLI for PacBio HiFi (knock-knock process-sample <base_dir> hbb_ghasemi <sample> --stages preprocess,align,categorize) and a custom Python driver that instantiates Nanopore Experiment with the HDR categorizer for Oxford Nanopore (knock-knock's CLI does not natively dispatch the nanopore platform). For each of the four quadrants (PacBio HiFi × {TRAC, TRBC} and Nanopore × {TRAC, TRBC}), strategies were built from the truncated synthetic WT and HDR donor references using knock-knock build-strategies, with per-strategy targets.csv, sgRNAs.csv, donors.csv, and amplicon_primers.csv configurations (one row per primer, as required by knock-knock's parser). Because preliminary runs on the raw simulation FASTQs placed 11.7%–34.5% of reads in a single malformed layout: extra copy of primer category — a preprocessing artifact traced to incidental sequence similarity between the 24-nt PacBio SMRTbell adapter motif (5′-GTACTTCGTTCAGTTACGTATTGC-3′) and the interior of the TRBC forward primer — SMRTbell adapter motifs were exhaustively trimmed from both ends of every read using exact k-mer matching (k ≥ 13 for HiFi reads, k ≥ 11 for ONT reads to accommodate the higher base-error rate) prior to knock-knock processing. After trimming, the extra_copy_of_primer rate dropped 7×–241× across the four quadrants. All knock-knock runs were executed sequentially on a single CPU core under GNU /usr/bin/time -v to capture wall-clock time, peak resident set size, and CPU utilization, and the per-read calls in outcome_list.txt were mapped onto ALPINE's 13-class schema by the rules documented in compute_benchmark_metrics.py.

Classification accuracy was evaluated by comparing read classification class with ground truth class. For ALPINE, benchmarking was straightforward as the tool's classification categories directly correspond to simulated template classes. For knock-knock, its broader output categories were mapped to the corresponding simulated ground truth labels, where knock-knock’s "complex mis-integration" encompassed ITR-mediated integration, cross-target HDR and ITR templates; "deletion in donor" mapped to truncated HDR templates; "WT" encompassed both wild-type and SNP categories; “insertion” class covered both large and small insertions. Performance metrics including per-class precision, recall, F1-scores, and overall accuracy were calculated using standard confusion matrix analysis across the 12 template categories. All customized scripts used for simulated dataset generation and benchmark are available at <https://github.com/Maggi-Chen/ALPINE-manuscript-analysis>.

**Supplementary Note 5: Public dataset analysis**

Public datasets were obtained from NCBI SRA via ENA's filereport API rather than the SRA Toolkit. For each BioProject, the per-run manifest (run accession, sample title, library strategy, instrument model, read count, FASTQ FTP URL, and FASTQ file size) was retrieved with the query [https://www.ebi.ac.uk/ena/portal/api/filereport?accession=<PRJNA-id>&result=read_run&fields=run_accession,sample_title,library_strategy,instrument_model,read_count,fastq_ftp,fastq_bytes&format=tsv](https://www.ebi.ac.uk/ena/portal/api/filereport?accession=%3cPRJNA-id%3e&result=read_run&fields=run_accession,sample_title,library_strategy,instrument_model,read_count,fastq_ftp,fastq_bytes&format=tsv), and the FASTQ files were downloaded directly via wget from the resolved fastq_ftp URLs. PacBio HiFi accessions were verified to be CCS reads (despite the SRA _subreads.fastq.gz filename convention) by inspecting per-read length distributions (CCS shows tight amplicon-size IQR of <50 bp) and quality-score saturation at Phred Q93 (~); subreads, which would have shown 5–50 kb length variance and Q30-capped scores, were not used.

ALPINE was run with multi-platform launcher with command “python launcher.py -s sample_sheet_PRJNA913199.txt --project_id alpine-revision-public-datasets --reffile reference_PRJNA913199.fasta --config config.tsv --primer_1 CCTGAGACTTCCACACTGAT --primer_2 AATAAGGAGAAGATATGCTT --primer_check_length 100 --min_qual 20 --platform SevenBridges --five_prime_HA_seq CCTGAGACTTCCACACTGATGCAATCATTCGTCTGTTTCCCATTCTAAACTGTACCCTGTTACTTATCCCCTTCCTATGACATGAACTTAACCATAGAAAAGAAGGGGAAAGAAAACATCAAGCGTCCCATAGACTCACCCTGAAGTTCTCAGGATCCACGTGCAGCTTGTCACAGTGCAGCTCACTCAGTGTGGCAAAGGTGCCCTTGAGGTTGTCCAGGTGAGCCAGGCCATCACTAAAGGCACCGAGCACTTTCTTGCCATGAGCCTTCACCTTAGGGTTGCCCATAACAGCATCAGGAGTGGACAGATCCCCAAAGGACTCAAAGAACCTCTGGGTCCAAGGGTAGACCACCAGCAGCCTAAGGGTGGGAAAATAGACCAATAGGCAGAGAGAGTCAGTGCCTATCAGAAACCCAAGAGTCTTCTCTGTCTCCACATGCCCAGTTTCTATTGGTCTCCTTAAACCTGTCTTGTAACCTTGATACCAACCTGCCCAGGGCCTCACCACCAACTTCATCCACGTTCACCTTGCCCCACAGGGCAG --three_prime_HA_seq TAACGGCAGACTTCTCCTCAGGAGTCAGATGCACCATGGTGTCTGTTTGAGGTTGCTAGTGAACACAGTTGTGTCAGAAGCAAATGTAAGCAATAGATGGCTCTGCCCTGACTTTTATGCCCAGCCCTGGCTCCTGCCCTCCCTGCTCCTGGGAGTAGATTGGCCAACCCTAGGGTGTGGCTCCACAGGGTGAGGTCTAAGTGATGACAGCCGTACCTGTCCTTGGCTCTTCTGGCACTGGCTTAGGAGTTGGACTTCAAACCCTCAGCCCTCCCTCTAAGATATATCTCTTGGCCCCATACCATCAGTACAAATTGCTACTAAAAACATCCTCCTTTGCAAGTGTATTTACGTAATATTTGGAATCACAGCTTGGTAAGCATATTGAAGATCGTTTTCCCAATTTTCTTATTACACAAATAAGAAGTTGATGCACTAAAAGTGGAAGAGTTTTGTCTACCATAATTCAGCTTTGGGATATGTAGATGGATCTCTTCCTGCGTCTCCAGAATATGCAAAATACTTACAGGACAGAATGGATGAAAACTCTACCTCGGTTCTAAGCATATCTTCTCCTTATTT”.

Knock-knock on PRJNA913199 PacBio HiFi: Knock-knock v0.8.2 was run on the 34 PacBio HiFi samples of PRJNA913199 using the full-genome workflow against pre-built hg38 indices (genomic flank for the amplicon was auto-extracted by build-strategies based on BLAST hits of the amplicon primers; reference orientation: HBB forward strand). The per-sample knock-knock strategy targeted the HBB E7V cleavage site with sgRNA CTTGCCCCACAGGGCAGTAA and amplicon primers CCTGAGACTTCCACACTGAT (forward) / AATAAGGAGAAGATATGCTT (reverse); the donor sequence was a 2,112 bp HBB-pUC19-GFP knock-in cassette reconstructed from Addgene plasmid pCR2086 (HA-L 540 bp, 1,151 bp GFP-tagged corrective payload, HA-R 421 bp). Because PacBio reads from this study carried a uniform ~30 bp SMRTbell adapter offset at each end that prevented knock-knock's HDR categorizer from anchoring its primer search, 30 bp were trimmed from each end of every read prior to processing using trim_smrtbell.py (the same trimmer used on the simulation benchmark, with the offset adapted for this library). Knock-knock was invoked per sample as knock-knock process-sample <base_dir> hbb_ghasemi <SRR_accession> under GNU /usr/bin/time -v to capture per-sample wall time, peak RAM, and CPU utilization. Per-read calls in outcome_list.txt were aggregated by category and sub-category and compared against ALPINE's per-sample classification of the same FASTQs.

CRISPResso2 on PRJNA913199 Illumina: CRISPResso2 (v2.2.x, conda-installed in our “biotools” environment) was run on the 43 paired-end Illumina samples of the same study, which target the identical HBB E7V locus and represent the short-read counterpart of the PacBio HiFi data. For each sample, CRISPResso -r1 <R1.fastq.gz> -r2 <R2.fastq.gz> -a <amplicon> -g <sgRNA> -an HBB -gn hsHBB -n <sample_name> -o <out_dir> --trim_sequences --n_processes 1 --suppress_report was invoked with the 382 bp HBB amplicon and the HBB E7V sgRNA. The --trim_sequences flag enables CRISPResso2's built-in adapter trimmer (Trimmomatic). Per-sample modification rates (HDR%, NHEJ%, mixed) were extracted from each output's CRISPResso_quantification_of_editing_frequency.txt and aggregated into crispresso2_per_sample_summary.csv for comparison with ALPINE's classification of the matched PacBio HiFi samples. We note that knock-knock and CRISPResso2 were run on different sequencing platforms of the same study (knock-knock on the long-read PacBio cohort, CRISPResso2 on the short-read Illumina cohort) because each tool is designed for its respective read-length regime; the two platforms target the same HBB E7V edit and so provide complementary cross-platform validation rather than a head-to-head comparison on identical reads.

Merged and per-sample output files of ALPINE, knock-knock, and CRISPResso2 were analyzed and visualized using customized scripts available at <https://github.com/Maggi-Chen/ALPINE-manuscript-analysis>.


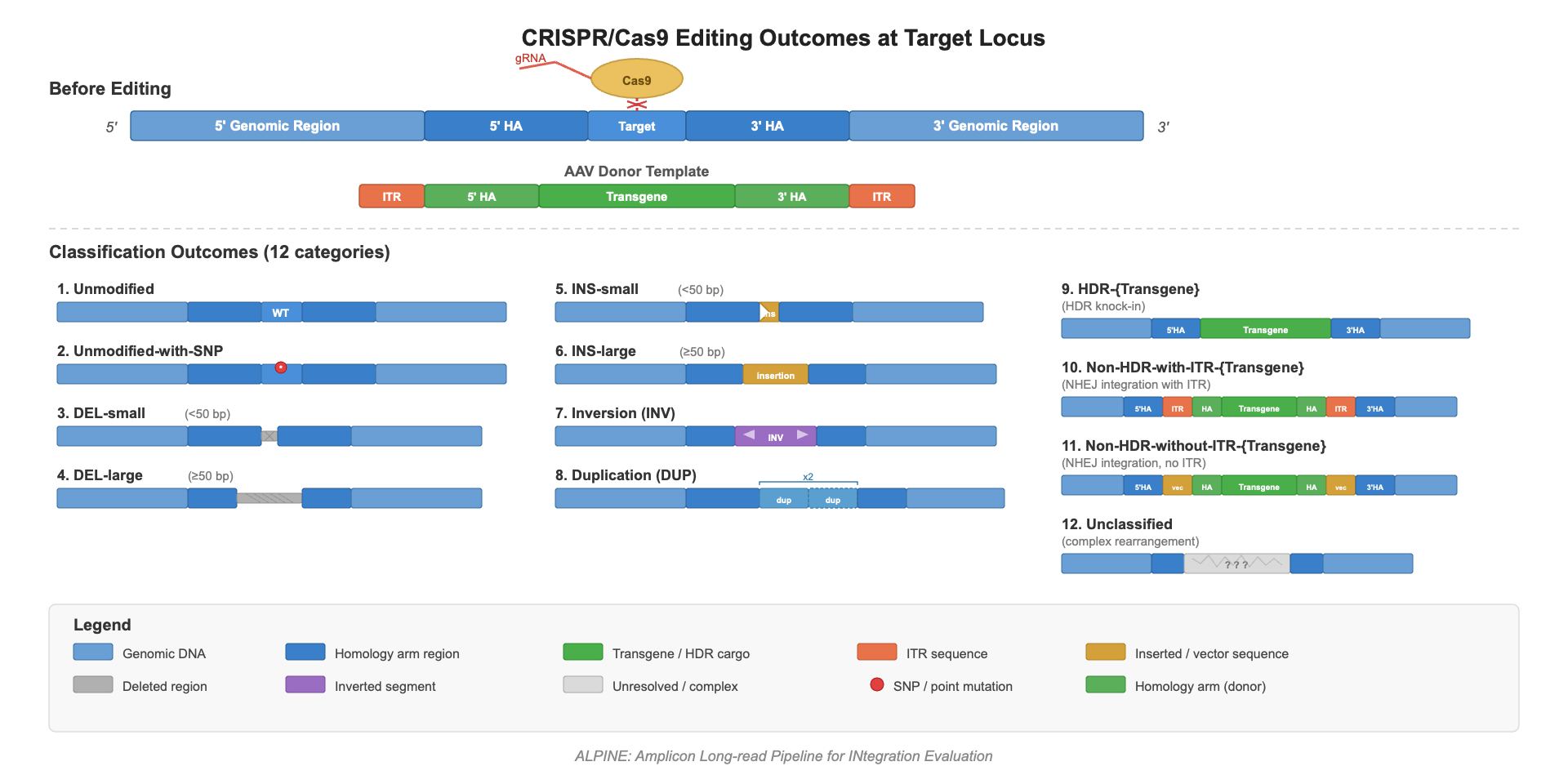


**Figure S1.** Cartoon schematic of CRISPR/Cas9 editing. Various genomic outcomes at the target site, including unmodified, unmodified-with-SNP, small and large deletions, small and large insertions, inversions, duplications, HDR knock-in, non-HDR vector insertion with and without ITR, and unclassified reads.


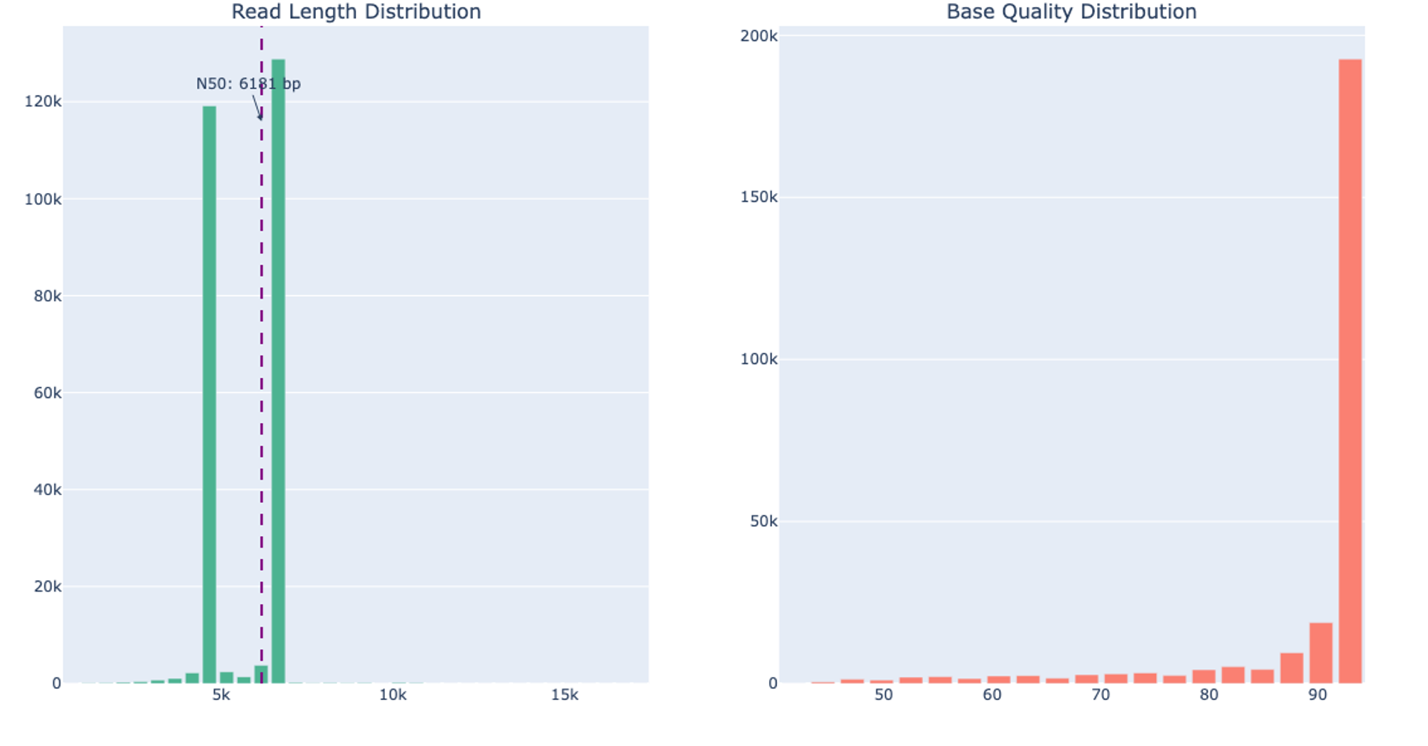


**Figure S2.** Representative read length and base quality distribution histograms from quality-control assessment.

**Figure S3.** Read classification algorithm flowchart showing the decision logic used to assign reads to variant categories based on alignment to wild-type, HDR, and AAV integration reference sequences, including three re-alignment modules and the patcher rescue step.

**Figure S4.** Representative pie chart showing the distribution of variant categories in a gene-edited sample.


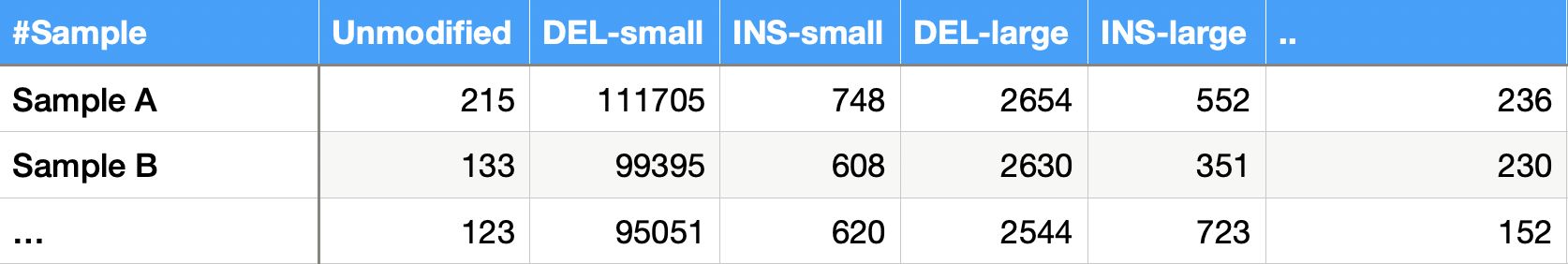


**Figure S5.** Example merged count table summarizing variant frequencies across multiple samples.

**Figure S6.** Accuracy benchmark of ALPINE and knock-knock in simulated dual-target PacBio HiFi and ONT datasets. HDR, ITR, and truncated HDR represent integration events with CD19 transgene integration into TRAC or CD22 transgene integration into TRBC. Cross-target HDR and cross-target ITR represent cross-contamination events with CD22 transgene sequences appearing in TRAC samples or CD19 transgene sequences appearing in TRBC samples. TRAC/TRBC Integration panels include HDR and ITR-related categories; TRAC/TRBC Variants panels include genomic variants and wild-type sequences at the cleavage sites.

| **Table S1. Simulated PacBio HiFi dataset benchmark - TRAC site** | | | | | | | | | |
| --- | --- | --- | --- | --- | --- | --- | --- | --- | --- |
| Tool | Template | Reads | Class | TP | FP* | FN | Precision* | Recall | F1 |
| ALPINE | HDR | 5000 | HDR-CD19 | 4,658 | 0 | 342 | 100.00 | 93.16 | 96.46 |
|  | ITR | 10000 | NonHDR-with-ITR-CD19 | 9,999 | 0 | 1 | 100.00 | 99.99 | 99.99 |
|  | Truncated HDR | 5000 | NonHDR-without-ITR-CD19 | 4,900 | 343 | 100 | 93.46 | 98.00 | 95.68 |
|  | Cross-Target-HDR | 5000 | HDR-CD22 | 4,860 | 0 | 140 | 100.00 | 97.20 | 98.58 |
|  | Cross-Target -ITR | 5000 | NonHDR-with-ITR-CD22 | 5,000 | 0 | 0 | 100.00 | 100.00 | 100.00 |
|  | Wild type | 5000 | Unmodified | 4,472 | 465 | 528 | 90.58 | 89.44 | 90.01 |
|  | SNP | 5000 | Unmodified-with-SNP | 4,460 | 21 | 540 | 99.53 | 89.20 | 94.08 |
|  | large DEL | 5000 | DEL-large | 4,949 | 87 | 51 | 98.27 | 98.98 | 98.62 |
|  | small DEL | 5000 | DEL-small | 4,910 | 565 | 90 | 89.68 | 98.20 | 93.75 |
|  | large INS | 5000 | INS-large | 4,774 | 183 | 226 | 96.31 | 95.48 | 95.89 |
|  | small INS | 5000 | INS-small | 4,667 | 543 | 333 | 89.58 | 93.34 | 91.42 |
|  | - | 0 | Unclassified/Other | 0 | 144 | 0 | 0.00 | 0.00 | 0.00 |
|  | Total integration** | 30000 |  | 29,417 | 343 | 583 | 98.85 | 98.06 | 98.45 |
|  | Total | 60000 |  | 57,649 | 2,351 | 2,351 | 96.08 | 96.08 | 96.08 |
| knock-knock | HDR | 5000 | HDR | 4,974 | 0 | 26 | 100.00 | 99.48 | 99.74 |
|  | ITR | 10000 | complex misintegration | 9,995 | 734 | 5 | 96.43 | 99.95 | 98.16 |
|  | Truncated HDR | 5000 | deletion in donor | 4,570 | 0 | 430 | 100.00 | 91.40 | 95.51 |
|  | Cross-Target-HDR | 5000 | complex misintegration | 4,997 | 734 | 3 | 96.43 | 99.94 | 98.16 |
|  | Cross-Target-ITR | 5000 | complex misintegration | 4,854 | 734 | 146 | 96.43 | 97.08 | 96.76 |
|  | Wild type | 5000 | WT | 4,887 | 468 | 113 | 94.89 | 97.74 | 96.29 |
|  | SNP | 5000 | WT | 3,804 | 468 | 1,196 | 94.89 | 76.08 | 84.45 |
|  | large DEL | 5000 | deletion >=50 nt | 4,722 | 81 | 278 | 98.31 | 94.44 | 96.34 |
|  | small DEL | 5000 | deletion <50 nt | 4,519 | 52 | 481 | 98.86 | 90.38 | 94.43 |
|  | large INS | 5000 | insertion | 0 | 144 | 5,000 | 64.53 | 0.00 | 0.00 |
|  | small INS | 5000 | insertion | 262 | 144 | 4,738 | 64.53 | 5.24 | 9.69 |
|  | - | 0 | uncategorized/Other | 0 | 10,937 | 0 | 0.00 | 0.00 | 0.00 |
|  | Total integration** | 30000 |  | 29,390 | 734 | 610 | 97.56 | 97.97 | 97.76 |
|  | Total | 60000 |  | 47,584 | 12,416 | 12,416 | 79.31 | 79.31 | 79.31 |

TP, true positive; FP, false positive; FN, false negative.

*FP and Precision are based on knock-knock's classification class where many-to-one mapping occurs. Affected categories: ITR, Cross-target-HDR, and Cross-target-ITR are all classified as "complex misintegration"; large and small insertions are classified as "insertion"; wild-type and SNP are classified as "WT". For categories mapped to the same knock-knock class, FP represents all false positives assigned to that knock-knock class, and precision represents the overall precision of that knock-knock class. Recall is always calculated based on simulation ground truth categories.

**Total integration sums all integration categories, including HDR, ITR, Truncated HDR, Cross-Target-HDR, and Cross-Target ITR.

| **Table S2. Simulated PacBio HiFi dataset benchmark - TRBC site** | | | | | | | | | |
| --- | --- | --- | --- | --- | --- | --- | --- | --- | --- |
| Tool | Template | Reads | Class | TP | FP* | FN | Precision* | Recall | F1 |
| ALPINE | HDR | 5000 | HDR-CD22 | 4,897 | 0 | 103 | 100.00 | 97.94 | 98.96 |
|  | ITR | 10000 | Non-HDR-with-ITR-CD22 | 9,999 | 0 | 1 | 100.00 | 99.99 | 99.99 |
|  | Truncated HDR | 5000 | Non-HDR-without-ITR-CD22 | 5,000 | 104 | 0 | 97.96 | 100.00 | 98.97 |
|  | Cross-Target-HDR | 5000 | HDR-CD19 | 4,872 | 0 | 128 | 100.00 | 97.44 | 98.70 |
|  | Cross-Target -ITR | 5000 | Non-HDR-with-ITR-CD19 | 5,000 | 0 | 0 | 100.00 | 100.00 | 100.00 |
|  | Wild type | 5000 | Unmodified | 4,251 | 562 | 749 | 88.32 | 85.02 | 86.64 |
|  | SNP | 5000 | Unmodified-with-SNP | 4,193 | 31 | 807 | 99.27 | 83.86 | 90.92 |
|  | large DEL | 5000 | DEL-large | 4,930 | 96 | 70 | 98.09 | 98.60 | 98.34 |
|  | small DEL | 5000 | DEL-small | 4,899 | 1,175 | 101 | 80.66 | 97.98 | 88.48 |
|  | large INS | 5000 | INS-large | 4,710 | 42 | 290 | 99.12 | 94.20 | 96.60 |
|  | small INS | 5000 | INS-small | 4,633 | 470 | 367 | 90.79 | 92.66 | 91.72 |
|  | - | 0 | Unclassified/Other | 0 | 136 | 0 | 0.00 | 0.00 | 0.00 |
|  | Total integration** | 30000 |  | 29,768 | 104 | 232 | 99.65 | 99.23 | 99.44 |
|  | Total | 60000 |  | 57,384 | 2,616 | 2616 | 95.64 | 95.64 | 95.64 |
| knock-knock | HDR | 5000 | HDR | 4,932 | 0 | 68 | 100.00 | 98.64 | 99.32 |
|  | ITR | 10000 | complex misintegration | 9,923 | 807 | 77 | 96.05 | 99.23 | 97.61 |
|  | Truncated HDR | 5000 | deletion in donor | 4,695 | 0 | 305 | 100.00 | 93.90 | 96.85 |
|  | Cross-Target-HDR | 5000 | complex misintegration | 4,963 | 807 | 37 | 96.05 | 99.26 | 97.63 |
|  | Cross-Target -ITR | 5000 | complex misintegration | 4,744 | 807 | 256 | 96.05 | 94.88 | 95.46 |
|  | Wild type | 5000 | WT | 4,764 | 572 | 236 | 93.67 | 95.28 | 94.47 |
|  | SNP | 5000 | WT | 3,696 | 572 | 1,304 | 93.67 | 73.92 | 82.63 |
|  | large DEL | 5000 | deletion >=50 nt | 4,578 | 87 | 422 | 98.14 | 91.56 | 94.73 |
|  | small DEL | 5000 | deletion <50 nt | 4,261 | 186 | 739 | 95.82 | 85.22 | 90.21 |
|  | large INS | 5000 | insertion | 0 | 149 | 5,000 | 19.46 | 0.00 | 0.00 |
|  | small INS | 5000 | insertion | 36 | 149 | 4,964 | 19.46 | 0.72 | 1.39 |
|  | - | 0 | uncategorized/Other | 0 | 11,607 | 0 | 0.00 | 0.00 | 0.00 |
|  | Total integration** | 30000 |  | 29,257 | 807 | 743 | 97.32 | 97.52 | 97.42 |
|  | Total | 60000 |  | 46,592 | 13,408 | 13,408 | 77.65 | 77.65 | 77.65 |

TP, true positive; FP, false positive; FN, false negative.

*FP and Precision are based on knock-knock's classification class where many-to-one mapping occurs. Affected categories: ITR, Cross-target-HDR, and Cross-target-ITR are all classified as "complex misintegration"; large and small insertions are classified as "insertion"; wild-type and SNP are classified as "WT". For categories mapped to the same knock-knock class, FP represents all false positives assigned to that knock-knock class, and precision represents the overall precision of that knock-knock class. Recall is always calculated based on simulation ground truth categories.

**Total integration sums all integration categories, including HDR, ITR, Truncated HDR, Cross-Target-HDR, and Cross-Target ITR.

| **Table S3. Simulated ONT dataset benchmark - TRAC site** | | | | | | | | | |
| --- | --- | --- | --- | --- | --- | --- | --- | --- | --- |
| Tool | Template | Reads | Class | TP | FP* | FN | Precision* | Recall | F1 |
| ALPINE | HDR | 5000 | HDR-CD19 | 4,999 | 64 | 1 | 98.74 | 99.98 | 99.35 |
|  | ITR | 10000 | NonHDR-with-ITR-CD19 | 9,896 | 0 | 104 | 100.00 | 98.96 | 99.48 |
|  | Truncated HDR | 5000 | NonHDR-without-ITR-CD19 | 4,993 | 41 | 7 | 99.19 | 99.86 | 99.52 |
|  | Cross-Target-HDR | 5000 | HDR-CD22 | 4,992 | 0 | 8 | 100.00 | 99.84 | 99.92 |
|  | Cross-Target -ITR | 5000 | NonHDR-with-ITR-CD22 | 5,000 | 0 | 0 | 100.00 | 100.00 | 100.00 |
|  | Wild type | 5000 | Unmodified | 1,111 | 129 | 3,889 | 89.60 | 22.22 | 35.61 |
|  | SNP | 5000 | Unmodified-with-SNP | 2,320 | 1,318 | 2,680 | 63.77 | 46.40 | 53.72 |
|  | large DEL | 5000 | DEL-large | 4,750 | 97 | 250 | 98.00 | 95.00 | 96.48 |
|  | small DEL | 5000 | DEL-small | 4,892 | 4,011 | 108 | 54.95 | 97.84 | 70.37 |
|  | large INS | 5000 | INS-large | 4,720 | 76 | 280 | 98.42 | 94.40 | 96.37 |
|  | small INS | 5000 | INS-small | 4,624 | 1,896 | 376 | 70.92 | 92.48 | 80.28 |
|  | - | 0 | Unclassified/Other | 0 | 71 | 0 | 0.00 | 0.00 | 0.00 |
|  | Total integration** | 30000 |  | 29,880 | 105 | 120 | 99.65 | 99.60 | 99.62 |
|  | Total | 60000 |  | 52,297 | 7,703 | 7,703 | 87.16 | 87.16 | 87.16 |
| knock-knock | HDR | 5000 | HDR | 4,825 | 0 | 175 | 100.00 | 96.50 | 98.22 |
|  | ITR | 10000 | complex misintegration | 9,833 | 2,424 | 167 | 88.11 | 98.33 | 92.94 |
|  | Truncated HDR | 5000 | deletion in donor | 2,847 | 0 | 2,153 | 100.00 | 56.94 | 72.56 |
|  | Cross-Target-HDR | 5000 | complex misintegration | 4,900 | 2,424 | 100 | 88.11 | 98.00 | 92.79 |
|  | Cross-Target -ITR | 5000 | complex misintegration | 3,232 | 2,424 | 1,768 | 88.11 | 64.64 | 74.57 |
|  | Wild type | 5000 | WT | 2,717 | 261 | 2,283 | 94.81 | 54.34 | 69.08 |
|  | SNP | 5000 | WT | 2,049 | 261 | 2,951 | 94.81 | 40.98 | 57.22 |
|  | large DEL | 5000 | deletion >=50 nt | 2,949 | 64 | 2,051 | 97.88 | 58.98 | 73.61 |
|  | small DEL | 5000 | deletion <50 nt | 2,991 | 895 | 2,009 | 76.97 | 59.82 | 67.32 |
|  | large INS | 5000 | insertion | 0 | 604 | 5,000 | 21.15 | 0.00 | 0.00 |
|  | small INS | 5000 | insertion | 162 | 604 | 4,838 | 21.15 | 3.24 | 5.62 |
|  | - | 0 | uncategorized/Other | 0 | 19,247 | 0 | 0.00 | 0.00 | 0.00 |
|  | Total integration** | 30000 |  | 25,637 | 2,424 | 4,363 | 91.36 | 85.46 | 88.31 |
|  | Total | 60000 |  | 36,505 | 23,495 | 23,495 | 60.84 | 60.84 | 60.84 |

TP, true positive; FP, false positive; FN, false negative.

*FP and Precision are based on knock-knock's classification class where many-to-one mapping occurs. Affected categories: ITR, Cross-target-HDR, and Cross-target-ITR are all classified as "complex misintegration"; large and small insertions are classified as "insertion"; wild-type and SNP are classified as "WT". For categories mapped to the same knock-knock class, FP represents all false positives assigned to that knock-knock class, and precision represents the overall precision of that knock-knock class. Recall is always calculated based on simulation ground truth categories.

**Total integration sums all integration categories, including HDR, ITR, Truncated HDR, Cross-Target-HDR, and Cross-Target ITR.

| **Table S4. Simulated ONT dataset benchmark - TRBC site** | | | | | | | | | |
| --- | --- | --- | --- | --- | --- | --- | --- | --- | --- |
| Tool | Template | Reads | Class | TP | FP* | FN | Precision* | Recall | F1 |
| ALPINE | HDR | 5000 | HDR-CD22 | 4,999 | 79 | 1 | 98.44 | 99.98 | 99.21 |
|  | ITR | 10000 | Non-HDR-with-ITR-CD22 | 9,893 | 0 | 107 | 100.00 | 98.93 | 99.46 |
|  | Truncated HDR | 5000 | Non-HDR-without-ITR-CD22 | 4,998 | 29 | 2 | 99.42 | 99.96 | 99.69 |
|  | Cross-Target-HDR | 5000 | HDR-CD19 | 4,995 | 0 | 5 | 100.00 | 99.90 | 99.95 |
|  | Cross-Target -ITR | 5000 | Non-HDR-with-ITR-CD19 | 5,000 | 0 | 0 | 100.00 | 100.00 | 100.00 |
|  | Wild type | 5000 | Unmodified | 1,126 | 178 | 3,874 | 86.35 | 22.52 | 35.72 |
|  | SNP | 5000 | Unmodified-with-SNP | 2,218 | 1,260 | 2,782 | 63.77 | 44.36 | 52.32 |
|  | large DEL | 5000 | DEL-large | 4,746 | 87 | 254 | 98.20 | 94.92 | 96.53 |
|  | small DEL | 5000 | DEL-small | 4,890 | 4,480 | 110 | 52.19 | 97.80 | 68.06 |
|  | large INS | 5000 | INS-large | 4,668 | 51 | 332 | 98.92 | 93.36 | 96.06 |
|  | small INS | 5000 | INS-small | 4,610 | 1,619 | 390 | 74.01 | 92.20 | 82.11 |
|  | - | 0 | Unclassified/Other | 0 | 74 | 0 | 0.00 | 0.00 | 0.00 |
|  | Total integration** | 30000 |  | 29,885 | 108 | 115 | 99.64 | 99.62 | 99.63 |
|  | Total | 60000 |  | 52,143 | 7,857 | 7,857 | 86.91 | 86.91 | 86.91 |
| knock-knock | HDR | 5000 | HDR | 4,796 | 0 | 204 | 100.00 | 95.92 | 97.92 |
|  | ITR | 10000 | complex misintegration | 9,706 | 2,283 | 294 | 88.72 | 97.06 | 92.70 |
|  | Truncated HDR | 5000 | deletion in donor | 3,154 | 1 | 1,846 | 99.97 | 63.08 | 77.35 |
|  | Cross-Target-HDR | 5000 | complex misintegration | 4,859 | 2,283 | 141 | 88.72 | 97.18 | 92.76 |
|  | Cross-Target -ITR | 5000 | complex misintegration | 3,384 | 2,283 | 1,616 | 88.72 | 67.68 | 76.78 |
|  | Wild type | 5000 | WT | 3,104 | 377 | 1,896 | 93.54 | 62.08 | 74.63 |
|  | SNP | 5000 | WT | 2,352 | 377 | 2,648 | 93.54 | 47.04 | 62.60 |
|  | large DEL | 5000 | deletion >=50 nt | 2,828 | 58 | 2,172 | 97.99 | 56.56 | 71.72 |
|  | small DEL | 5000 | deletion <50 nt | 2,895 | 894 | 2,105 | 76.41 | 57.90 | 65.88 |
|  | large INS | 5000 | insertion | 0 | 421 | 5,000 | 8.68 | 0.00 | 0.00 |
|  | small INS | 5000 | insertion | 40 | 421 | 4,960 | 8.68 | 0.80 | 1.46 |
|  | - | 0 | uncategorized/Other | 0 | 18,849 | 0 | 0.00 | 0.00 | 0.00 |
|  | Total integration** | 30000 |  | 25,899 | 2,283 | 4,101 | 91.90 | 86.33 | 89.03 |
|  | Total | 60000 |  | 37,118 | 22,882 | 22,882 | 61.86 | 61.86 | 61.86 |

TP, true positive; FP, false positive; FN, false negative.

*FP and Precision are based on knock-knock's classification class where many-to-one mapping occurs. Affected categories: ITR, Cross-target-HDR, and Cross-target-ITR are all classified as "complex misintegration"; large and small insertions are classified as "insertion"; wild-type and SNP are classified as "WT". For categories mapped to the same knock-knock class, FP represents all false positives assigned to that knock-knock class, and precision represents the overall precision of that knock-knock class. Recall is always calculated based on simulation ground truth categories.

**Total integration sums all integration categories, including HDR, ITR, Truncated HDR, Cross-Target-HDR, and Cross-Target ITR.

**Figure S7. Comparison of gene editing outcome classification by three computational tools.** Stacked bar charts comparing the proportion of different outcome categories as classified by ALPINE (top), knock-knock (center), and CRISPResso2 (bottom) at HBB editing site. Other category includes unclassified (ALPINE) and uncategorized (knock-knock) and all other categories not listed here. Unmodified, unmodified K562 cells without plasmid; Untreated, modified and untreated; UV, modified with UV-light treatment only; EtOH, modified with UV + ethanol solvent; 4M/10M/200M, modified with UV + 4uM/10uM/200uM psoralen treatment; 0.01X/0.1X/0.3X/1X/3X/10X/30X, modified with UV + relative psoralen densities. No significant difference was found between CRISPResso2 UV group and treated groups using Welch's two-sample t-test (p-values: EtOH = 0.094, 0.01X = 0.190, 0.03X = 0.095, 0.1X = 0.311, 0.3X = 0.473, 1X = 0.073, 3X = 0.056, 10X = 0.457, 30X = 0.576).

**Figure S8.** Correlation analysis of gene editing outcome classification between ALPINE and knock-knock. Scatter plots comparing category proportions detected by ALPINE (y-axis) versus knock-knock (x-axis) for small deletions, large deletions, insertions, HDR, and wild-type outcomes. Left panels show all samples; right panels show modified samples only (excluding unmodified controls). Blue lines show linear regression with 95% confidence intervals. Pearson correlation coefficients (r) displayed in each panel.

**Figure S9. Detection rate of 9bp-deletion by ALPINE and knock-knock.** Bar plots comparing proportion of reads containing 9bp deletion reported by ALPINE and knock-knock across different experimental treatments. Colored bars represent mean 9bp-deletion proportion for each treatment group, with error bars indicating standard error. Individual black dots show raw data points for each sample. Sample sizes: Unmodified, n=4; all other treatment groups, n=6.

**Figure S10. Proportion of plasmid integration reported by ALPINE and knock-knock.** Bar plots comparing proportion of reads classified as by HDR or Non-HDR-without-ITR categories by ALPINE, and classified as HDR, incomplete HDR, or donor fragment categories by knock-knock. Bars are colored by treatment group. n.s., not significant; ***, p<0.01 from Wilcoxon test compared to Untreated group.

**Figure S11. Alignment of reads containing plasmid integration.** IGV screenshots of integration category reads classified by both ALPINE and knock-knock (**A**) and by ALPINE only (**B**). Reads are aligned against HDR template plasmid sequence (left homology arm + payload + right homology arm). Locations of homology arms are marked at bottom panel of each screenshot.

| **Table S5. Summary of gene edits** | | | | | | | | |
| --- | --- | --- | --- | --- | --- | --- | --- | --- |
|  | **TransGene A** | | | | **TransGene B** | | | |
| **Sample** | **HDR KI** | **Indels** | **Non-HDR** | **Other** | **HDR KI** | **Indels** | **Non-HDR** | **Other** |
| **Sample_1** | 69.711% | 23.006% | 6.771% | 0.511% | 54.811% | 41.884% | 3.138% | 0.167% |
| **Sample_2** | 61.102% | 30.044% | 8.197% | 0.657% | 58.974% | 38.280% | 2.502% | 0.244% |
| **Sample_3** | 83.982% | 12.365% | 3.239% | 0.414% | 52.307% | 45.330% | 2.180% | 0.183% |
| **Sample_4** | 80.041% | 15.764% | 3.799% | 0.395% | 51.576% | 46.308% | 1.920% | 0.196% |
| **Sample_5** | 81.302% | 15.295% | 2.942% | 0.461% | 47.804% | 49.973% | 2.042% | 0.180% |

HDR KI includes HDR category. Indels includes DEL-small, DEL-large, INS-small, and INS-large categories. Non-HDR includes Non-HDR-w/o-ITR-A, Non-HDR-w/o-ITR-B, Non-HDR-w/-ITR-A, and Non-HDR-w/-ITR-B categories. Other includes INV, DUP, SNP, and Unmodified categories.


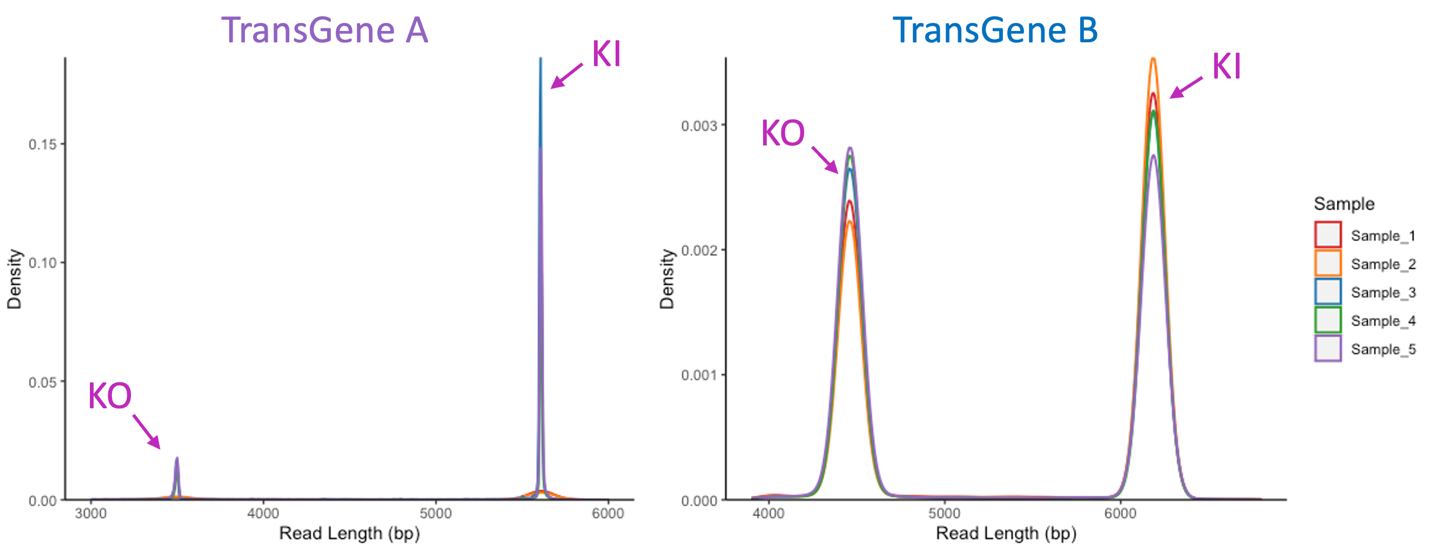


**Figure S12. Read length distributions of five samples.** Peaks for knock-out (KO) and knock-in (KI) are marked with arrowheads in the histogram. KO peak includes reads from Indels and Other categories. KI peak includes reads from HDR KI and Non-HDR categories.

| **Table S6. Runtime and peak RAM usage** | | | | | |
| --- | --- | --- | --- | --- | --- |
| Dataset | Read/sample | Tool | Runtime  (wall clock) | Peak RAM  (GB) |  |
| Simulation | 60,000 | ALPINE per-sample | 7m 32 s | 1.35 |  |
|  |  | knock-knock per-sample | 23m 1s | 0.77 |  |
| PRJNA913199 | 7,205 to 42,733 | ALPINE per-sample | 4m 36s | 1.35 |  |
|  |  | ALPINE launcher (all samples) | 10m 40s | - |  |
|  |  | knock-knock per-sample | 7m 47s | 0.61 |  |

ALPINE per-sample run was benchmarked on the Seven Bridges Genomics platform using c4.2xlarge instances. ALPINE launcher run was benchmarked on a single core of an AWS EC2 r5.8xlarge instance (32 vCPU, 256 GB RAM). Knock-knock per-sample run was benchmarked on a single core of an AWS g6e.8xlarge instance (32 vCPU, 256 GB RAM).
